# Differences in cause and 12-month follow-up outcome of parkinsonian symptoms in depressed older adults treated with antipsychotics: a case series

**DOI:** 10.1186/s12888-021-03298-9

**Published:** 2021-06-03

**Authors:** Anastasios Politis, Nikolaos Kokras, Michael Souvatzoglou, Kostas Siarkos, Panagiotis Toulas, Constantin Potagas, Theodoros Hatzipanagiotou, Georgios Limouris, Panagiotis Alexopoulos

**Affiliations:** 1 Charing Cross Hospital, Department of Neurosurgery, Imperial College London, London, UK; 2 First Department of Psychiatry, Eginition Hospital; Department of Pharmacology, Medical School, National and Kapodistrian University of Athens, Athens, Greece; 3 Nuclear Medicine Division, Radiology First Department, “Aretaieion” Hospital, Medical School, National and Kapodistrian University of Athens, Athens, Greece; 4 First Department of Psychiatry, Eginition Hospital, Medical School, National and Kapodistrian University of Athens, Athens, Greece; 5 Research Unit of Radiology, Second Department of Radiology, National and Kapodistrian University of Athens and Bioiatriki, Athens, Greece; 6 First Department of Neurology, Eginition Hospital, Medical School, National and Kapodistrian University of Athens, Athens, Greece; 7 Department of Nuclear Medicine, Metropolitan Hospital, Neo Faliro, Pireas, Greece; 8 Department of Psychiatry, Patras University Hospital, Faculty of Medicine, School of Health Sciences, University of Patras, 26504 Rion, Patras, Greece; 9 Department of Psychiatry and Psychotherapy, Klinikum rechts der Isar, Faculty of Medicine, Technical University of Munich, Munich, Germany

**Keywords:** Drug induced parkinsonism, Parkinson’s disease, Vascular parkinsonism, Idiopathic tremor, Case series

## Abstract

**Background:**

Parkinsonian symptoms are common adverse effects of antipsychotics. Older adults are particularly vulnerable to drug-induced parkinsonism. Nonetheless, parkinsonian symptoms in seniors treated with antipsychotics cannot be straightforwardly attributed to antipsychotic medication. A comprehensive diagnostic workup is necessary in many cases in order to shed light on the cause of such symptoms in this patient population.

**Case series:**

Eight cases of hospitalized depressed older adults with parkinsonian symptoms, who were treated for at least one year with antipsychotics, are reported. Based on neurological consultation, structural brain imaging and Ioflupane (I-123) dopamine transporter (DAT) single photon emission computerized tomography (SPECT), Parkinson’s disease was diagnosed in one case, idiopathic tremor in another, vascular parkinsonism in another one, while in another individual parkinsonian symptoms persisted at 12-month post-discharge follow-up even though his/her symptoms were classified as drug-induced on discharge. In four patients, parkinsonian symptoms were definitely drug-induced and no movement disturbances were reported at follow-up.

**Conclusions:**

Differences in the cause and outcome of parkinsonian symptoms in seniors treated with antipsychotics merit systematic and in-depth study considering the therapeutic and prognostic implications of an accurate detection of the cause of such symptoms. Familiarizing clinical psychiatrists with these differences could pave the way towards approaching seniors with severe, atypical and/or persistent parkinsonian symptoms in a more individualized diagnostic and therapeutic manner, and towards more cautious prescribing of antipsychotics in this age group.

## Background

Antipsychotic augmentation is not a rare strategy in the pharmacological treatment of depression in older adults. Τhe prevalence of clinically relevant depressive symptoms in seniors is higher than 15%, while the prevalence of major depression exceeds 5% [1, 2]. Antipsychotics augment antidepressants in 12–32% of older adults with depression [3–6], albeit in most cases in an off-label manner [7]. Antipsychotics contribute not only to treating psychotic symptoms in severe depression with psychotic features [8], but also to depressive symptom improvement, since they enhance monoaminergic neurotransmission [9].

Seniors are particularly vulnerable to side effects of antipsychotic agents, such as parkinsonian symptoms. Both typical antipsychotics, which mainly block D2 receptors, and to a lesser degree atypical ones, blocking both D2 and serotonin 5-HT2A receptors, can lead to parkinsonian symptoms [10, 11]. The annual prevalence of drug-induced parkinsonian symptoms approximates 3% and the annual incidence rate is higher than 3 per 100,00 person-years [12, 13]. Seniors are more vulnerable to parkinsonian symptoms compared to younger patients due to age- related changes in the brain’s response to antipsychotics, diminished drug metabolizing capacity, alterations in blood-brain barrier, as well as drug-drug interactions in an age group burdened by polypharmacy [14]. In addition, age- related, still presymptomatic, neurodegenerative (e.g. Lewy-bodies, nigral cell degeneration) [12, 15] and/or vascular brain changes [16] should be considered as factors crucially implicated in the high susceptibility of seniors treated with antipsychotics to develop severe and persistent parkinsonian symptoms which cannot be unambiguously classified into diagnostic categories. Structural imaging and Ioflupane (I-123) dopamine transporter (DAT) single photon emission computerized tomography (DaTSAN) are useful tools in shedding light on such brain changes and assisting the differential diagnosis [17, 18]. Normal DaTSCAN-imaging supports diagnosis of a condition not involving nigrostriatal neurodegeneration such as Alzheimer’s disease, essential tremor or drug-induced parkinsonism and hence a different therapeutic approach [19]. DaTSCAN sensitivity is estimated to be 78–100% and specificity 70–100% for differentiating neurodegenerative versus non-neurodegenerative parkinsonism [20].

Here, we report on eight hospitalized depressed older adults with parkinsonian symptoms who were treated for at least 12 months prior to their hospital admission with antipsychotics. The presented case series highlights the different causes and 12-month post-discharge follow-up outcomes of such symptoms, despite the initial assessment on admission that they were drug-induced.

## Case series presentation

The case series consists of five women and three men older than 59 years who were admitted between 2012 and 2017 to the old age psychiatry division of the Eginition Hospital in Athens because of severe depressive symptoms and consecutively agreed to undergo brain SPECT with 123I-FP-CIT (DaTSCAN, GE Healthcare). The brief, focused neurological examination on admission revealed parkinsonian symptoms, which had been developed during exposure to antipsychotic treatment (Table 1). On admission, each patient was on antipsychotic medication that had not been changed in the twelve months prior to his/her hospital admission (Table 1). Five patients were on atypical and three on typical antipsychotics; four were on serotonin and norepinephrine reuptake inhibitors, two on selective serotonin reuptake inhibitor and four were treated with mirtazapine. Except for one patient, who had received the diagnosis of bipolar disorder many years before current hospital admission, all others had been diagnosed with unipolar depression. Of note, depression had occurred among four patients for the first time at an age greater than or equal to 65 years with no previous history of depression.

**Table 1:** Demographic and clinical data, mental disorder history and medication of older adults with depression and parkinsonian symptoms on hospital admission

Case No	Age range	Education (in years)	Neuropsychological assessment at admission		Duration of current depressive syndrome (in years)	Past mental disorder diagnoses	Age of onset of the first mental disorder episode	Medication on admission (in mg/day)	Comorbidities
			Geriatric depression scale score	Mini mental state examination score					
1	71–85	12	12	21	1	Μajor depression	69	Amisulpiride (100), venlafaxine (75), mirtazapine (30), ferrous sulfate, nifedipine (40)	Anaemia, hypertension
2	71–85	14	14	27	1	Severe depressive episode with psychotic symptoms	72	Risperidone (3), sertraline (200 mg), mirtazapine (30), lorazepam (2.5), irbesartan (150), metoprolol (100)	Hypertension, hypothyreroidism
3	56–70	14	14	28	1	Severe depressive episode with psychotic symptoms	57	Olanzapine (5), mirtazapine (45), alprazolam (3), flunitrazepam (1), biperiden (4)	None
4	56–70	14	15	20	1	Moderate to severe depressive episode	36	Haloperidol (10), venlafaxine (150), lorazepam (7.5), biperiden (6), pindolol (5), rosuvastatin (10), atorvastatin (10)	Hypertension, dislipidemia
5	71–85	8	12	27	1	Moderate to severe depressive episode with psychotic symptoms	77	Haloperidol (10), sertraline (100), bromazepam (3), amiloride hydrochloride/hydrochlorothiazide (5/50)	Hypertension
6	71–85	12	14	23	1	Bipolar disorder	54	Risperidone (3), mirtazapine (45), gabapentin (600), clonazepam (2), baclofen (20), metformin (2000), rosuvastin (20), bisoprolol fumarate (5), Levothyroxine sodium (125 μg)	Hypertension, diabetes mellitus, coronary heart disease, dyslipidemia, hypothyroidism
7	71–85	6	15	27	1	Severe depressive episode with psychotic symptoms	76	Haloperidol (20), venlafaxine (150), pramipexol (0.18), quinapril (20), rabeprazole (10)	Hypertension
8	56–70	12	13	19	1	Moderate to severe depressive episodes	55	Quetiapine (100), duloxetine (30), pregabalin (75), levodopa/benserazide (200/50)	Hypertension

The diagnostic workup of depressive symptoms included a history from the patient and from an informant; psychiatric examination; laboratory screening and the administration of the 30-point Mini-Mental State Examination (MMSE) [21] and the Geriatric depression scale-15 [22]. Geriatric depression scale score was in all cases higher than 11, highlighting the severity of depressive symptoms (Table 1). In four patients MMSE score was lower than 24 points, pointing to major neurocognitive disorder [21], even though the magnitude of the detected cognitive deficits was supposed to be at least partially potentiated by the depressive syndrome [23].

Patients were diagnosed with antipsychotic- induced parkinsonism according to the International Statistical Classification of Diseases and Related Health Problems version 10 (ICD-10) diagnostic criteria [24]. Severity of parkinsonian symptoms was graded with the Simpson-Angus scale (SAS), being an established instrument of neuroleptic-induced parkinsonism [25]. The instrument is a 10-item rating scale that measures gait (hypokinesia) with one item, rigidity with six items, whilst three additional items assess glabella tap, tremor and salivation, respectively. Items are rated from 0 to 4 points, yielding a total body score divided by 10, and considered normal up to 0.3. It was performed in all cases by the same psychiatrist (KS). Apart from one case, in all other patients total scores on SAS exceeded 0.5 (Table 2).

**Table 2:** Parkinsonian symptoms of depressed older adults

Case No	Detected movement disturbances on admission	Simpson-Angus Scale										
		Total score	Gait	Arm dropping	Shoulder shaking	Elbow rigidity	Wrist rigidity	Leg pendulousness	Head dropping	Glabella tap	Tremor	Salivation
1	Parkinsonism	0.7	1	1	0	1	2	0	1	0	1	0
2	Parkinsonism	0.9	2	1	0	0	2	1	0	1	2	0
3	Parkinsonism	0.6	1	1	1	0	1	1	0	0	1	0
4	Parkinsonism	1.4	1	1	1	1	2	1	1	1	3	1
5	Parkinsonism	1.6	2	2	2	2	1	2	1	1	3	0
6	Parkinsonism	1.3	2	2	1	2	2	1	1	1	1	0
7	Parkinsonism	0.3	0	1	0	0	1	0	0	0	1	0
8	Parkinsonism	1.1	1	1	1	1	2	1	1	1	2	0

**Fig. 1:**
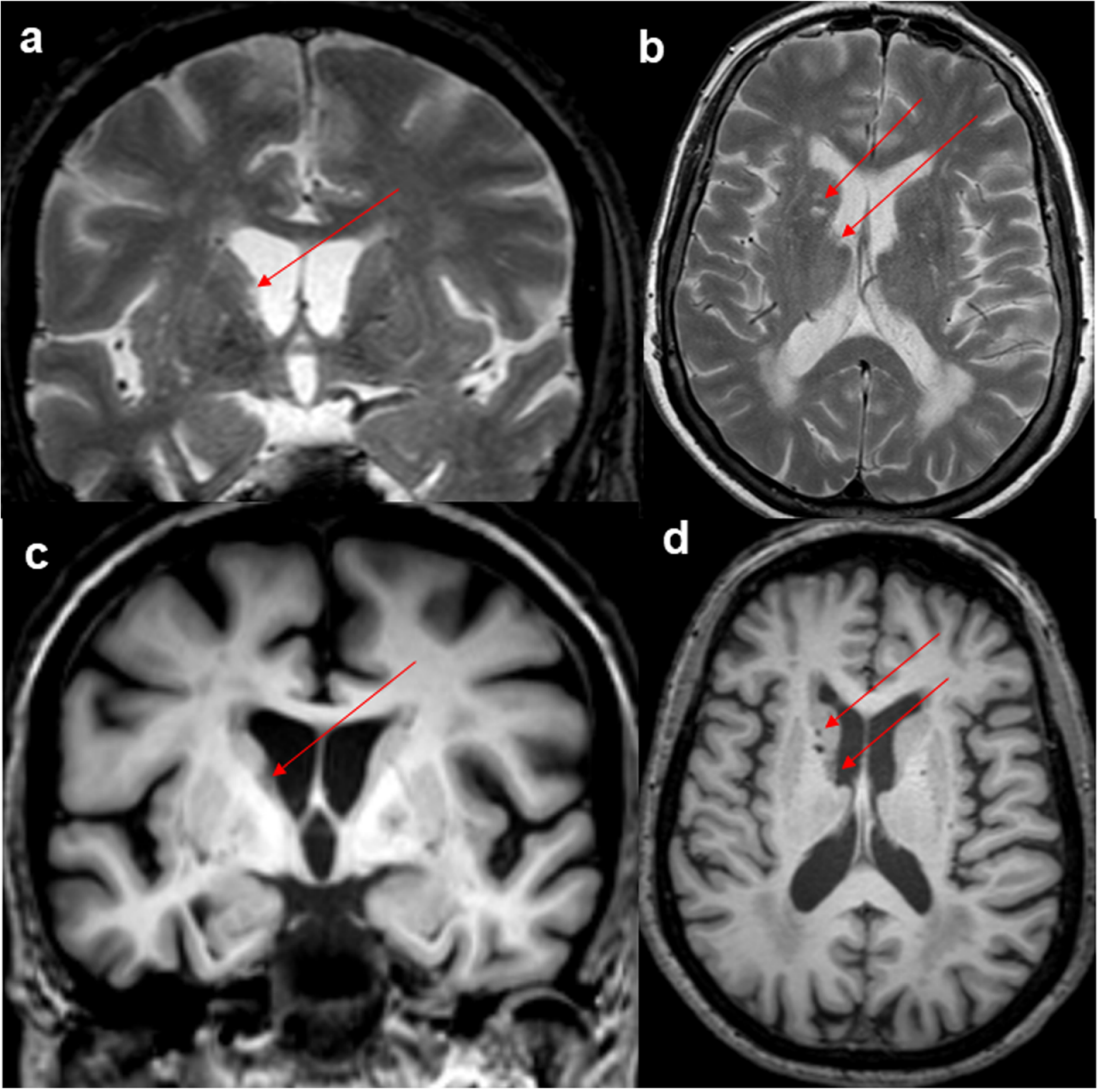
Brain MRI (3 T) of case 8. Lacunar lesions in superior-anterior-medial surface of right thalamus in the proximity inferior surface of caudal nucleus (arrows) (**A**: coronal T2* weighted image and **B**: axial T2 weighted image). Microvascular leukoencephalopathy (Fazekas grade 3). No hemosiderin accumulation in basal ganglia in T2* weighted images. No medial temporal lobe atrophy (**C** and **D**: coronal and axial T1 weighted images)

Brain MRI (3 T) was performed during the hospitalization period. It included T1 weighted sequences on coronal, sagittal and axial planes (or isotropic T1 images), T2 weighted sequences, T2 Fluid Attenuation Inversion Recovery (FLAIR) images and Diffusion Weighted Imaging (DWI). All MRI scans were evaluated by an experienced neuroradiologist (PT). White matter lesions and vascular disease were evaluated using the Fazekas scale on T2-FLAIR sequences [26]. This scale ranges from 0 (no white matter lesions) to 3 (large confluent areas of white matter lesions) [27]. Based on the Medial Temporal Lobe Atrophy (MTA) -Scheltens Score, MTA was classified using a scale from 0 (no atrophy) to 4 (maximum atrophy) [28]. Unfortunately, MRI imaging was not conducted in two cases due to MRI contraindications (Table 3). Slightly increased signal in T2-weighted images in white matter was observed in four cases and beginning confluent lesions were detected in one case, while in case 8 basal ganglia ischemic lesions were bilaterally observed in addition to large confluent white matter lesions (Fig. 1). Mild or moderate MTA was detected in all cases with available MRI data (Table 3).

**Table 3:** Brain imaging findings of older adults with depression and parkinsonian symptoms

Case No	Structural brain MR imaging		DaT scan*			
	MTA-Scheltens Score**	White matter lesions/Fazekas Score***	Specific Binding Index right		Benamer’s criteria****	
			Right	Left	Rater 1	Rater 2
1	2	1	2.17	2.10	0	0
2	1	2	2.05	2.12	1	1
3	1	0	2.04	2.03	0	0
4	No atrophy / Computerized tomography (CT) scan		2.03	2.02	0	1
5	2	1	2.02	2.06	0	0
6	No atrophy / Computerized tomography (CT) scan		2.00	1.88	2	3
7	2	1	1.85	2.07	1	1
8	2	Bilateral basal ganglia ischemic lesions/3	1.51	1.67	1	1

* iofluropane iodine-123 (DAT) single-photon emission computed tomography imaging (SPECT)

**Medial Temporal Lobe Atrophy (MTA) -Scheltens Score ranging from 0 (no atrophy) to 4 (severe atrophy)

***The 4-point Fazekas score is a whole brain scale: 0: No lesion or a single punctuate white matter lesion; 1: multiple punctuate lesions; 2: Beginning confluent lesions; 3: large confluent lesions

**** Benamer’s criteria: 0: preserved and largely symmetrical striatal tracer uptake; 1: asymmetric uptake with normal or almost normal putamen activity in one hemisphere, and with a more marked reduction in the contralateral putamen; 2: significant bilateral reduction in putamen uptake with activity confined to the caudate nuclei; 3: virtually absent uptake bilaterally affecting both putamen and caudate nuclei

**Fig. 2:**
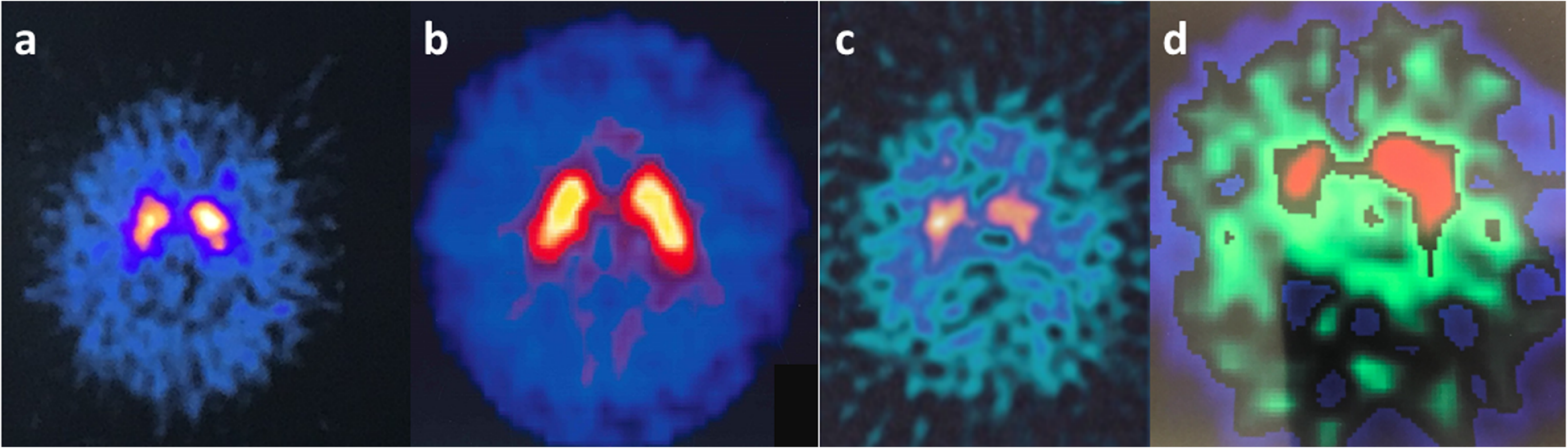
^123^I-Ioflupane SPECT imaging, a measure of dopamine transporter density, demonstrates in case 2 normal radiotracer uptake or very mild hypoactivity in right caudate (**A**); in case 3 normal radiotracer uptake bilaterally (**B**); in case 6 bilateral striatal hypoactivity (**C**) and in case 8 bilateral hypoactivity with a more marked reduction in right striatum(**D**)

Due to the severity and persistence of parkinsonian symptoms, despite cessation of antipsychotic medication in four cases or switching to other antipsychotic agents in the rest of cases, the assessment of the basal ganglia’s presynaptic dopamine function was recommended. Patients agreed and underwent DaTSCAN. DaTSCAN scintigraphy acquisition and processing methodology as well as the analysis method of the striatal uptake ratios of DaTSCAN images have been previously depicted in detail [29, 30]. Visual analysis of the data was performed by two nuclear medicine specialists who were blind to patients’ clinical status. Tracer uptake patterns were classified according to the Benamer’s criteria [31] as normal (preserved and largely symmetrical striatal tracer uptake), or abnormal grade 1 in case of asymmetric uptake with normal or almost normal putamen activity in one hemisphere, and with a more marked reduction in the contralateral putamen, or abnormal grade 2, if significant bilateral reduction in putamen uptake with activity confined to the caudate nuclei was detected, or abnormal grade 3 in cases with virtually absent uptake bilaterally affecting both putamen and caudate nuclei [32]. The semi-quantitative analysis and calculation (cnts/pixel) of striatal uptake was based on manually drawn, irregular (almost ellipsoid) regions of interest (ROIs), encompassing the entire corpus striatum, and square ROIs in areas corresponding to the occipital cortex in the three consecutive ‘best’ subsets of a transaxial slice through the central striatum of the DaTSCAN image. The specific radiotracer binding ratios of the striatum were calculated from its mean counts per pixel vs those of the occipital cortex as reference [specific binding index in the striatum (S.B.I. ≥ 2.0)] = value for the striatum divided to the value for the occipital cortex reference [29]. According to the visual analysis three patients had a normal study; οne had a marginally asymmetrical tracer uptake; three patients had an abnormal tracer uptake with moderately asymmetric dopaminergic loss and one patient exhibited a significant bilateral reduction in tracer uptake. Consistent with previous reports, the clinical agreement between the two raters was 75% [33, 34]. The specific radiotracer binding indexes point to one case with symmetrically abnormal radiotracer uptake and to two cases with unilateral abnormal uptake, whilst in all other patients the uptake was within the normal range (Table 3) (Fig. 2). For the sake of clarity of presentation, the eight cases are presented in all tables in a descending order regarding the semi-quantitative calculation of tracer uptake in right striatum.

Based on clinical data, GDS- and MMSE results and imaging findings and in accordance with ICD-10 diagnostic criteria mental disorder- and neurological diagnoses were established [24]. Depressive syndromes were classified as organic mood disorder in four cases and as severe depressive episode of recurrent depressive disorder in the other four. Based on neurological consultation, the diagnosis of Parkinson’s disease was established only in one case, whilst one patient with normal striatal tracer uptake was diagnosed with idiopathic tremor and case 8 with vascular parkinsonism. In all other cases parkinsonian symptoms were attributed to the administration of antipsychotics. Mild cognitive impairment due to Alzheimer’s disease was diagnosed in two cases. On discharge, no patient was treated with typical antipsychotics, while in four cases no antipsychotics were administered at all. Antiparkinsonian drugs were initiated during hospitalization or continued in six patients (Table 4). Interestingly, apart from one case, the number of prescribed drugs on discharge was lower in five cases or in two cases equal to that of agents with which patients were treated on admission.

**Table 4:** Neurological and mental disorder diagnoses and medication on discharge and 12-month post-discharge follow-up outcome regarding movement disturbances and mental disorder- and neurological diagnoses

Case No	Hospitalization duration (in days)	Discharge neurological diagnosis	Discharge mental disorder diagnosis	Medication at discharge	12-month post-discharge follow-up outcome
1	45	Drug-induced parkinsonism Mild Cognitive Impairment due to Alzheimer’s disease	Organic mood disorder	Duloxetine (90), donepezil (5)	No movement disturbances Dementia due to Alzheimer’s disease
2	40	Idiopathic tremor	Severe depressive episode of recurrent depressive disorder	Quetapine (25), duloxetine (60), clonazepam (2), levothyroxine sodium (100 μg)	Idiopathic tremor
3	90	Drug-induced parkinsonism	Severe depressive episode of recurrent depressive disorder	Risperidone (4), paroxetine (20), mirtazapine (60), alprazolam (4), biperiden (4)	No movement disturbances
4	120	Drug-induced parkinsonism Tardive dyskinesia Mild Cognitive Impairment due to Alzheimer’s disease	Severe depressive episode of recurrent depressive disorder	Quetiapine (150), trazodone (50), mirtazapine (45), lorazepam (7.5), biperiden (2), pindolol (5)	Tardive dyskinesia Dementia due to Alzheimer’s disease
5	60	Drug-induced parkinsonism	Organic mood disorder	Quetiapine (50), duloxetine (90), lorazepam (0.5), levodopa/benserazide (100/25), amiloride, hydrochloride/hydrochlorothiazide (5/50)	Parkinsonism Persistent psychotic symptoms
6	35	Parkinson’s disease	Organic mood disorder	Escitalopram (40), lorazepam (1), levodopa/benserazide (600/150), amantadine (100)	Parkinsonism
7	100	Drug-induced parkinsonism	Severe depressive episode of recurrent depressive disorder	Duloxetine (60), mirtazapine (45), lorazepam (1), levodopa/benserazide (200/50)	No movement disturbances Dementia due to Alzheimer’s disease
8	40	Vascular parkinsonism Microvascular ischemic brain disease	Organic mood disorder	Duloxetine (30), rivastigmine (4.6), levodopa/benserazide (200/50), ilbesartan (150)	Parkinsonism Vascular dementia

For the 12-month post-discharge follow-up outcome assessment, a caregiver-based telephone interview format was employed. The interview was based on questions about movement disturbances and mental disorder- and neurological diagnoses. The individuals diagnosed on discharge with Parkinson’s disease, idiopathic tremor and vascular parkinsonism, respectively, still suffered from movement disturbances, as expected. In a patient who had been diagnosed on discharge with drug-induced parkinsonism, persistent movement disturbances were reported, despite the discontinuation of antipsychotic agents during hospitalization. In all other patients no parkinsonian symptoms were reported, in line with the discharge diagnosis of drug- induced parkinsonism. The two patients who received on discharge the diagnosis of mild cognitive impairment due to Alzheimer’s disease progressed to dementia and faced at follow-up difficulties with both complex and basic activities of daily living.

## Discussion and conclusions

The development of parkinsonian symptoms while being on antipsychotic medication does not straightforwardly substantiate the presence of drug-induced parkinsonism, especially in seniors. The presented case series, which in contrast to the heterogeneity of study samples of most past reports [35–37], focuses exclusively on older adults with depression treated with antidepressants and antipsychotics, clearly points to a diagnostic riddle in this patient population.

Treatment with antipsychotics can induce parkinsonian symptoms, uncover subclinical degenerative and/or vascular striatal brain pathologies, or lead to the exacerbation of previously mildly manifest parkinsonian symptoms [38]. Drug-induced parkinsonism is conceptualized as parkinsonism that occurs during drug exposure and resolves within six months after the withdrawal of the implicated agent [12], even though symptoms can persist beyond six to nine months cessation of the drug without evidence of impaired striatal dopamine transporter density [18]. Drug-induced parkinsonism may be distinguished from Parkinson’s disease, since contrary to the latter, drug-induced parkinsonism is characterized by bilateral and symmetric parkinsonism, with more prominent bradykinesia and rigidity. Nonetheless, relying exclusively on the clinical manifestation of parkinsonian symptoms in order to differentiate between drug-induced parkinsonism and other causes is not a reliable and feasible strategy particularly in seniors who commonly develop atypical symptom phenotypes [12]. Ιndeed, more than 30% of patients with drug-induced parkinsonism show asymmetric parkinsonism and tremor at rest [12, 38].

In older adults treated with antipsychotics, the puzzle of the genesis and clinical manifestation of parkinsonian symptoms are further perplexed by the heavy burden of coexisting brain pathologies (e.g. amyloidosis, cerebrovascular alterations, mitochondrial abnormalities) [39, 40]. The clinical expression of such brain pathologies may be potentiated or masked by a complex, dynamic interplay between the interacting co-pathologies and harmful and protective environmental factors (e.g. diet and lifestyle parameters, motor reserve) [41, 42]. Exposure to antipsychotic agents influences this interplay and may trigger the development of clinically evident parkinsonian symptoms. This dynamic equilibrium is mirrored in the complex relationships between DaTSCAN- and MRI- findings, clinical diagnoses and 12-month follow-up outcome (resolution vs. persistence of parkinsonian symptoms) in the presented eight cases. For instance, bilateral basal ganglia ischemic lesions seem to have increased the vulnerability of case 8 to the development of parkinsonian symptoms, even though he/she was treated with quetiapine, i.e. an atypical antipsychotic with low risk of aggravation of parkinsonism [12].

Deciphering the cause of parkinsonian symptoms in seniors treated with antipsychotics is crucial for designing the proper, individualized therapeutic plan. Prescribing antiparkinsonian drugs without an established diagnosis of Parkinson’s disease or even of a Parkinson-plus syndrome, which albeit responds in most cases poorly to current therapies compared to Parkinson’s disease [43], is not justifiable. Antiparkinsonian agents can lead to further exacerbation of psychotic symptoms (dopaminergic agents) or induce cognitive impairment or delirium (anticholinergic agents), whilst polypharmacy is related to adverse outcomes, including adverse drug events and disability in this age group [44, 45]. On the other hand, cessation of the assumed causative agent in order to shed light on the cause of the parkinsonian symptoms and distinguish between drug-induced parkinsonian symptoms and a movement disorder may not be practical in many cases, since depression in late life tends to be a persistent or recurrent and not rarely a therapy resistant condition [23], while persistence or paradoxical progressive deterioration of parkinsonism upon antipsychotic withdrawal have been described [30, 46]. Thus, a rigorous and comprehensive diagnostic workup of parkinsonian symptoms, including structural brain imaging and DaTSCAN, seems to be in cases with atypical, severe and/or persistent parkinsonian symptoms inevitable, in order to design justifiable, individualized treatment plans. In addition, such a diagnostic workup can be prospectively conducted in patients thought to be at high risk for parkinsonism because of age, family history or subthreshold parkinsonian symptoms [30], so that a vulnerable degenerated nigrostriatal tract is identified and considered in decision making about the treatment plan.

Despite the phenotypic homogeneity and the thorough and rigorous diagnostic workup of the considered patients, the presented case series has a number of limitations. Even though all patients suffered from severe depressive symptoms on admission, four were diagnosed at discharge with recurrent depressive disorder and four with organic mood disorder, while in one case the diagnosis of bipolar disorder had been established prior hospital admission. Moreover, the age of onset of the first mood disorder episode as well as performance on MMSE varied across the cases. Only four cases can be classified as suffering from late-onset depression. In four cases MMSE scores pointed to clinically significant abnormalities in cognitive function [21]. Taking into account the differences in symptoms and pathogenesis of late-onset depression compared to other forms of depression [23], the complex relationship between depression and cognitive impairment in seniors [47], as well as the higher vulnerability of older adults to side effects of antipsychotics, studies on parkinsonian symptoms with homogenous samples with regard to mental disorder diagnosis, age at symptom onset, cognitive status and antipsychotic medication are needed. In the present cases series, the follow-up assessment was conducted twelve months after hospital discharge, although drug-induced parkinsonian symptoms can persist longer than twelve months after cessation of the offending drug [12]. However, in general they resolve in six to nine months and 70% of patients usually recover within a few months after withdrawal of the causative drug [12, 36]. In addition, the diagnostic tools employed in the eight presented cases are characterized by shortcomings. For instance, the SAS has been criticized for overemphasizing body rigidity [48] and DaTSCAN interrater variability and inconsistencies between visual interpretation and classification based on quantitative radiotracer uptake calculation [49], which were also observed in cases presented here (Table 3), are further sources of uncertainty. Regarding the impact of DaTSCAN, the diagnosis itself is not exclusively driven by imaging findings, although physicians reported previously that DaTSCAN influences their final diagnosis, confidence in their diagnosis and management of the patient [50]. Of note, a case series including eight individuals does not suffice to generalize conclusions. Nonetheless, the observed differences in cause and 12-month follow-up outcome in this limited number of depressed older adults treated with antipsychotics point to the uncertainties related to the pathogenesis and development of parkinsonian symptoms in this clinical phenotype and age group and may fuel future research aiming to shed more light on a diagnostic riddle with therapeutic and prognostic implications.

In conclusion, detecting the cause of parkinsonian symptoms in seniors who are exposed to antipsychotics and subsequently taking appropriate action is a challenging task. Indeed, parkinsonian symptoms cannot be straightforwardly classified as drug-induced in older adults treated with antipsychotics. Age-related neurodegenerative and vascular brain changes may underlie parkinsonism and shape a terrain of unreliable clinical characteristics, ambiguous exam findings and in many cases hardly justifiable therapeutic choices. Comprehensive diagnostic workup is in cases with atypical, severe and/or persistent parkinsonian symptoms the only feasible strategy for developing proper treatment plans. The field of deciphering the cause of parkinsonian symptoms in seniors treated with antipsychotics merits a systematic and thorough study of possible clinical, imaging and outcome endophenotypes of such symptoms, so that in the future personalized and efficient therapeutic strategies can be designed. Familiarizing clinical psychiatrists with effective ways to cope with inconsistent and/or ambiguous imaging and clinical data and make good diagnostic and therapeutic decisions is another step towards this direction.

## Data Availability

All data analyzed during this study are included in this published article in tables or text.

## Abbreviations

5-HT2A receptor: 5-hydroxy-tryptamin-type 2A receptor
GDS: Geriatric depression scale
MMSE: Mini-mental state examination
SAS: Simpson-Angus scale
MRI: Magnetic resonance imaging
FLAIR: Fluid Attenuation Inversion Recovery
DWI: Diffusion weighted imaging
MTA: Medial temporal lobe atrophy
DaTSCAN: Ioflupane (I-123) dopamine transporter (DAT) single photon emission computerized tomography (SPECT)
SBI: specific binding index
ICD-10: International Statistical Classification of Diseases and Related Health Problems version 10
